# Specific Contributions of Ventromedial, Anterior Cingulate, and Lateral Prefrontal Cortex for Attentional Selection and Stimulus Valuation

**DOI:** 10.1371/journal.pbio.1001224

**Published:** 2011-12-27

**Authors:** Daniel Kaping, Martin Vinck, R. Matthew Hutchison, Stefan Everling, Thilo Womelsdorf

**Affiliations:** 1 Department of Biology, Centre for Vision Research, York University, Toronto, Ontario, Canada; 2 Department of Physiology and Pharmacology, University of Western Ontario, London, Ontario, Canada; 3 Cognitive and Systems Neuroscience Group, Center for Neuroscience, University of Amsterdam, Amsterdam, The Netherlands; 4 Robarts Research Institute, London, Ontario, Canada; University of Oxford, United Kingdom

## Abstract

Attentional control ensures that neuronal processes prioritize the most relevant stimulus in a given environment. Controlling which stimulus is attended thus originates from neurons encoding the relevance of stimuli, i.e. their expected value, in hand with neurons encoding contextual information about stimulus locations, features, and rules that guide the conditional allocation of attention. Here, we examined how these distinct processes are encoded and integrated in macaque prefrontal cortex (PFC) by mapping their functional topographies at the time of attentional stimulus selection. We find confined clusters of neurons in ventromedial PFC (vmPFC) that predominantly convey stimulus valuation information during attention shifts. These valuation signals were topographically largely separated from neurons predicting the stimulus location to which attention covertly shifted, and which were evident across the complete medial-to-lateral extent of the PFC, encompassing anterior cingulate cortex (ACC), and lateral PFC (LPFC). LPFC responses showed particularly early-onset selectivity and primarily facilitated attention shifts to contralateral targets. Spatial selectivity within ACC was delayed and heterogeneous, with similar proportions of facilitated and suppressed responses during contralateral attention shifts. The integration of spatial and valuation signals about attentional target stimuli was observed in a confined cluster of neurons at the intersection of vmPFC, ACC, and LPFC. These results suggest that valuation processes reflecting stimulus-specific outcome predictions are recruited during covert attentional control. Value predictions and the spatial identification of attentional targets were conveyed by largely separate neuronal populations, but were integrated locally at the intersection of three major prefrontal areas, which may constitute a functional hub within the larger attentional control network.

## Abstract 2

Functional clusters of neurons in the monkey prefrontal and anterior cingulate cortex are involved in guiding attention to the most valuable objects in a scene.

## Author Summary

To navigate within an environment filled with sensory stimuli, the brain must selectively process only the most relevant sensory information. Identifying and shifting attention to the most relevant sensory stimulus requires integrating information about its sensory features as well as its relative value, that is, whether it's worth noticing. In this study, we describe groups of neurons in the monkey prefrontal cortex that convey signals relating to the value of a stimulus and its defining feature and location at the very moment when attention is shifted to the stimulus. We found that signals conveying information about value were clustered in a ventromedial prefrontal region, and were separated from sensory signals within the anterior cingulate cortex and the lateral prefrontal cortex. The integration of valuation and other “top-down” processes, however, was achieved by neurons clustered at the intersection of ventromedial, anterior cingulate, and lateral prefrontal cortex. We conclude that valuation processes are recruited when attention is shifted, independent of any overt behavior. Moreover, our analysis suggests that valuation processes can bias the initiation of attention shifts, as well as ensure sustained attentional focusing.

## Introduction

Selective attention prioritizes the processing of behaviorally relevant stimuli, at the expense of processing irrelevant stimuli [1]. Identifying the relevance of a stimulus requires neuronal circuitry to signal its associated value or reward outcome, in a given context. Recent evidence suggests that brain circuitry learns and processes the values associated with stimuli automatically, effectively biasing attentional stimulus selection towards more valuable stimuli in our environments [2]–[4]. In addition to such an involuntary capture of attention, the associated value of a stimulus has been suggested to be a critical feature that guides voluntary top-down deployment of attention [5]. Consistent with this suggestion, top-down control of attention has been shown to be facilitated and slowed down when target and distracting stimuli, respectively, are associated with a higher positive value [4],[6]–[10]. These behavioral findings suggest that stimulus valuation processes are a fundamental component of attentional top-down control and are integrated with attentional rule information that specifies to which stimulus or location attention will be shifted in response to environmental cues [11],[12].

Our study aimed to elucidate how the processing and integration of stimulus-value associations and top-down, attentional rule information map onto specific subdivision within the prefrontal cortex (PFC). The PFC has been long thought to play a role in identifying relevant stimuli and shifting attention towards them [13]–[16], and its various subdivisions may contribute specific computations for integrating and resolving conflict of competing valuation signals and top-down attentional rule information. There is compelling evidence that valuation signals about stimuli in choice tasks are encoded within ventromedial PFC (vmPFC), orbitofrontal PFC, and anterior cingulate cortex (ACC) [10],[11],[17]–[28]. It is unknown how these stimulus valuation signals are recruited to guide covert shifts of attention that require the flexible trial-by-trial mapping of stimulus relevance to stimulus location. Such flexible attention shifts are known to be severely compromised following large lesions to the lateral prefrontal cortex (LPFC) that spare medial frontal and orbitofrontal cortices [29]–[31]. But the relative contributions of the ventral and dorsal subdivisions of the LFPC have remained unclear. Within ventrolateral PFC, a large proportion of neuronal responses depends on the task relevance and reward outcome associated with a stimulus [27],[32],[33], even when working memory demands are controlled for [34]. The dorsolateral portion of the LPFC likewise hosts neurons sensitive to the reward outcome associated with response targets [27],[35]–[38], but is more generally implicated in preventing interference from irrelevant, distracting stimuli during attentional control [29],[39],[40]. The control of interference includes processes with various labels such as filtering [41], biasing of competition [42], resolving of conflict [43], or gating of inputs [44] and is likewise not strictly associated with the dorsolateral PFC, but closely linked to neuronal circuitry within the ACC [43]. That the ACC plays a prominent role for attentional control processes has long been suggested, but its putative involvement for the control of interference or the integration of valuation signals during attentional control has been supported exclusively by human fMRI studies [11],[14],[44]–[48].

**Figure 1:**
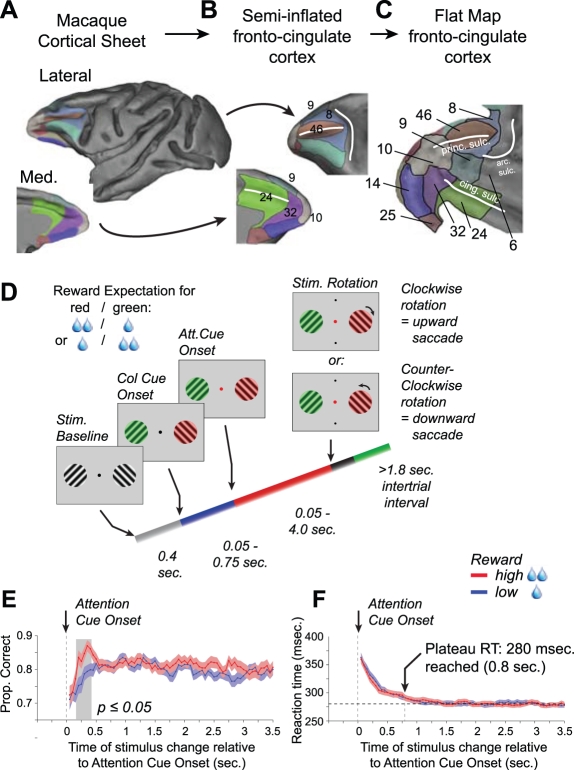
Fronto-cingulate anatomy, behavioral paradigm, and behavioral performance. (A) Lateral and medial view of the macaque cortex with color labeled anatomical subdivisions of the fronto-cingulate cortex following area definitions by Barbas and Zikopoulus [77] (see also Figure S1). (B) Fronto-cingulate subdivisions shown on partially inflated cortex. (C) A flat map representation of the fronto-cingulate cortex shown in panels A and B (obtained by cutting the inflated brain along the bottom and flipping it vertically, see Figure 2A,B), covering areas of ventromedial frontal cortex (areas 32, 25,14), anterior cingulate cortex (area 24) and lateral prefrontal cortex (areas 6, 8, 9, 10, 46). (D) *Behavioral paradigm*: Monkeys initiated a trial by directing and maintaining their gaze on a centrally presented fixation point. After 0.3 s two grating stimuli appeared drifting within two separate apertures (*Stim. Baseline*), and their respective colors changed to either red or green 0.4 s later (*Col. Cue Onset*). Within 0.05 to 0.75 s after this change in color of the grating, the central fixation point changed to either red or green, thereby cueing the monkeys to covertly shift attention towards the location where the color of the grating matched the color of the fixation point (*Att. Cue Onset*). In order to obtain a liquid reward, the monkeys had to detect a transient clock- or counterclockwise rotation of the cued target grating by making, respectively, up- and downward saccades towards a response target dot. This rotation of the cued target occurred at random times within 0.05–4 s, drawn from a uniform distribution. In half of the trials the distractor, i.e. the grating whose color did not match the color of the fixation point, rotated before the target (not shown). In a given trial, the red or green color of the cued target grating was associated with either a high or low liquid volume. This color-reward association changed every 30 correct trials. (E,F) The proportion of correct trials (E) and saccadic reaction times (F) for detecting the target's rotation, as a function of the time, relative to the attention cue onset, at which the target grating rotated. Red and blue lines correspond to, respectively, the “high-value” and “low-value” conditions. Color shading shows SEM.

To elucidate whether and how the processing and integration of stimulus values and attentional rule information actually maps onto specific subdivisions within the PFC around the time of covert attentional stimulus selection, we modified a conventional selective attention task that elicits clear attentional target selection signals in neurons with confined receptive fields in the frontal eye fields and in visual cortex [49],[50] by manipulating the attended target's location and associated value independently. We recorded from a large extent of the fronto-cingulate cortex of macaque monkeys (Figure 1A–C) and performed a reconstruction of the recording sites to topographically map the proportion of neurons that exhibited response modulations by target location, value, and the interaction between these two parameters.

## Results

### Behavioral Performance

We trained two macaque monkeys on a modified version of a conventional selective attention task (Figure 1D, see Material and Methods for details). Monkeys initiated a trial by directing and maintaining their gaze on a centrally presented fixation point. After 0.3 s, two black/white grating stimuli appeared drifting within two separate apertures, and their respective colors were changed to either red or green another 0.4 s later. Within 0.05 to 0.75 s after the change in grating color, the color of the central fixation point changed to either red or green, instructing the monkeys to covertly shift attention towards the location where the color of the grating matched the color of the fixation point. In order to obtain a liquid reward, the monkeys had to discriminate a smooth, transient clockwise or counterclockwise rotation of the cued target grating. The monkeys indicated the perceived rotation of the target grating (clockwise/counterclockwise) by making a saccade from the fixation point towards one of two response target dots presented vertically above or below the fixation point. The rotation direction (and hence the required saccade direction) was manipulated independently from the target grating's location and color. In half of the trials the distractor, i.e. the grating whose color did not match the color of the fixation point, rotated before the target. The rotation of the cued target grating and the distractor occurred at random times within 0.05–4 s drawn from a uniform probability distribution.

The volume of the liquid reward for correct responses was dependent on the grating color, with either red or green associated with a high volume, and the other color associated with a low volume. Color-reward associations were alternated every 30 correctly performed trials. In what follows, we will refer to the set of trials in which attention was cued to the grating that was associated with a high reward outcome as the *high-value condition*, and the set of trials in which attention was cued to the grating that was associated with a low reward outcome as the *low-value condition*.

The monkeys performed on average 78.6% (STD 10.0%) correct (76.6% and 83.9% for monkeys R and M, respectively) across 144 experimental sessions (78 and 66 with monkeys R and M, respectively) (Figure 1E). In trials in which the distractor rotated before the target, monkeys correctly ignored the unattended grating's rotation well above the 50% chance level (70.0%, STD 11.1%), compared to 87.3% (STD 9.7%) correct responses for trials in which there was no distractor change before the target rotation, consistent with previous reports of behavioral performance for a similar task [51]. As shown in Figure 1E, the association between target and outcome value modulated the behavioral accuracy to detect target changes occurring 0.15–0.4 s after the attention cue onset, with a significantly better performance for the high-value than the low-value condition (paired *t* test, *p*≤0.05).

Saccadic reaction times for the choice on the attentional target did not vary between the high-value and low-value condition, reaching an asymptotic level for choices made 0.8 s after attention cue onset (Figure 1F, see Text S1).

### Reconstruction of Recording Sites in Fronto-Cingulate Cortex

**Figure 2:**
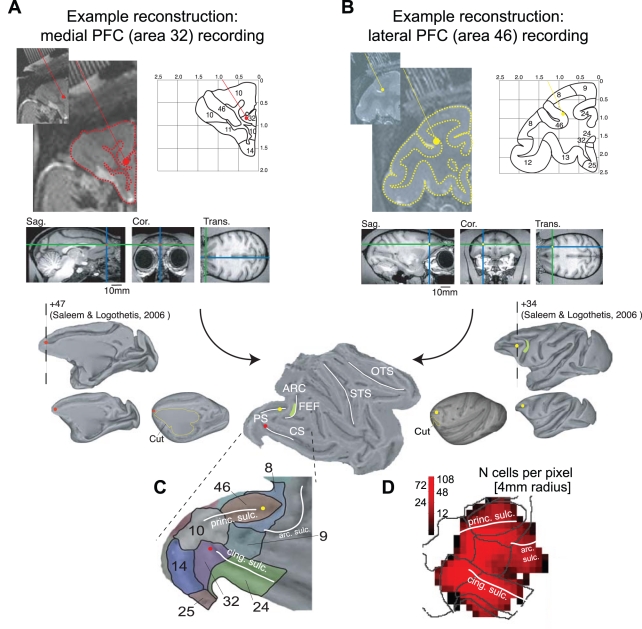
Example reconstructions of recording sites and spatial sampling density of the dataset. (A,B) Reconstruction of a medial PFC (area 32, A), and a lateral PFC (area 46, B) recording site started from the 7T anatomical MR, which was obtained with (iodine based) visualization of electrode trajectories within the electrode grid placed inside the recording chamber. The outline of the cortical folding was sketched on the coronal MR slice to ease identification of areas and landmarks according to standard brain atlases, and to place the depth of the electrode tip (red dot in A and yellow dot in B) with custom MATLAB code. The electrode position was then placed into a standardized macaque brain available in the MR Caret software package [52]. Caret allowed us to render the MR slice into a 3-D volume and to inflate the volume in order to finally cut (indicated as yellow line) the spherically inflated brain for representing it as 2-D flat map. (C) White lines on the flat map demarcate the principal sulcus (PS), the arcuate sulcus (ARC), and the cingulate sulcus (CS). The location of the FEF (frontal eye field) within the ARC is indicated by a green patch. Anatomical subdivisions in the fronto-cingulate cortex were visualized following the nomenclature from Barbas and Zikopoulus [77]. The area 32 and area 46 recording sites are visualized throughout the panels by a red and a yellow dot, respectively. (D) Number of cells recorded across areas overlaid on the contour of areal subdivisions (in grey) from the flat map in (C). For each pixel in the map, we counted the recorded cells within 4 mm radius (in steps of 2 mm).

We recorded the spiking activity of a total of 1,023 single neurons in the left hemispheres of two macaque monkeys during performance of the task. For each neuron, we reconstructed the recording sites based on high resolution, anatomical MRIs that visualized the electrode trajectories and provided landmarks to identify each site within a standardized macaque brain [52]. The sequence of reconstruction steps is shown in Figure 2A–C (see Materials and Methods for details). Projecting the reconstructed sites onto the two dimensional flat map shown in Figure 2C and counting the number of neurons around successive intersections of a regular grid that was spanned across the map revealed that we sampled neurons across the complete medial-to-lateral extent of the fronto-cingulate cortex (Figure 2D).

In the results that follow, we will only report analyses that were statistically corrected for uneven sampling, since the spatial sampling of neurons was uneven across the map, with up to 108 neurons at some pixels of the map and with the number of neurons sampled per pixel decreasing towards the borders of the map (i.e., the borders of the area covered by the recordings) (Figure 2D).

### Single Unit Examples of Spatial Attention Signals

**Figure 3:**
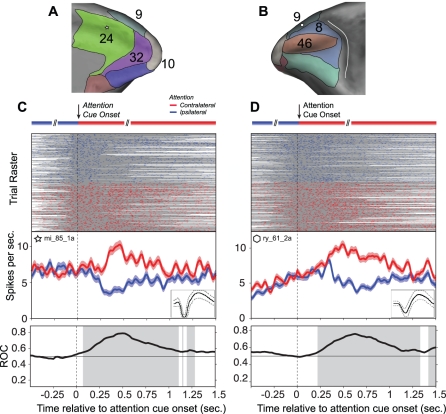
Example spike rasters. (A,B) The top panels show the semi-inflated medial (A) and lateral (B) view of the macaque fronto-cingulate cortex, with a white symbol indicating the site of two example neurons. (C) Attention-cue aligned spike raster across trials (top), spike density functions (middle), and ROC time course (bottom) for the example neuron from (A). The grey shading of the spike raster shows time epochs with identical visual stimulation, excluding contamination from the color onset of the peripheral grating stimuli (before cue onset) and any stimulus change occurring jittered in time after attention cue onset (see text for details). Color denotes spatial attention to the contralateral (red) and ipsilateral (blue) stimulus. Error bars denote SEM. Grey shading of the ROC time course highlight statistically significant time epochs based on permutation statistic on centered ±0.15 s windows. (D) Same format as (C), but for the example neuron in (B).

We focused our analyses around the time of the attention cue onset. Figure 3 shows the spike rasters and average firing rate evolution for two example neurons, separately for the *attend contralateral* and *attend ipsilateral* condition, demonstrating that we found reliable attention-cue induced neuronal signals that predicted whether monkeys were cued to shift attention to the contra- or the ipsilateral stimulus. The spike rasters also illustrate that our analysis included spikes only from time epochs with identical visual stimulation, i.e. void of the color onset of the peripheral stimuli, and that we limited our analysis until the time of the first stimulus rotation, which could be either of the distractor or of the target stimulus.

### Topography of Spatial Attention Effects and Outcome Value During Attention Shift

To quantify the spatial attention effect for a given neuron (i.e., the contrast in neuronal activity between the attend contralateral and the attend ipsilateral condition) over time, we performed a multifactorial ANOVA with value condition, attention condition, target grating color, and the interaction between value condition and attention condition as four independent explanatory variables (see Materials and Methods for details). To obtain a time-resolved analysis, we computed the ANOVAs for ±0.15 s time windows at successive time points (every 0.05 s) around the time of the attention cue onset. For each time point starting −0.25 s before the attention cue onset and ending 1.5 s after the attention cue onset, we identified whether neurons showed a significant spatial attention effect (p≤0.05, *F* test).

**Figure 4:**
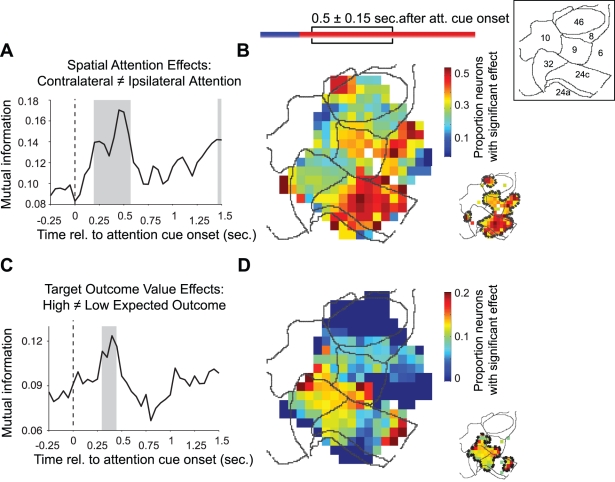
Topography of the effects of spatial attention and target value during the attention shift. (A) Mutual information between significant (*p*≤0.05) main effects of spatial attention (attend contra- versus ipsilateral stimulus) and location, as a function of the time relative to attention cue onset. The grey shading denotes time epochs with significant spatial clustering. (B) Fronto-cingulate map showing the distribution of the proportion of neurons with a significant spatial attention effect at 0.5±0.15 s after attention cue onset. The spatial areas with a larger proportion of significant neurons than expected by probability are highlighted by the black contours in the small map on the bottom right (see Materials and Methods for details). Each spatial map is overlaid by black contours, demarcating the area boundaries for the fronto-cingulate subdivisions as shown in Figures 1C and 2E. Area labels for each subdivision are indicated by the small inlet on the top-right of the figure. (C–D) Same format as (A,B), but now showing the mutual information between significant effects for target value (attention to target with high versus low expected outcome) and location.

We then tested, for each time point, whether the significant spatial attention effects of neurons clustered in space. As a first step, we quantified the mutual information between location (where a neuron fell into any of the 2-D pixels (“bins”) as in Figure 2D, i.e. the location variable was a bin number) and spatial attention selectivity, which was treated as a binary variable (i.e., 0/1 for non-significant/significant). Mutual information is defined as the difference between unconditional (in our case, ignoring attention or value condition) and conditional (in our case, conditional on attention or value condition) entropy of (i.e., the uncertainty about) the (binary) spatial attention selectivity. We used the mutual information measure to test whether neurons showing a significant spatial attention effect were more likely to be recorded at similar locations on the flat map, compared to the null hypothesis of a random, uniform spatial distribution of spatial attention effects (see Materials and Methods for details). Figure 4A shows the attention cue aligned evolution of the mutual information between location and spatial attention effect, illustrating that the amount of spatially specific clustering rose following cue onset, first reached statistical significance at 0.2 s (*p*≤0.05, *t* test), and peaked at 0.45 s after cue onset. This result demonstrates that we were able to predict whether a neuron had a significant attention effect by using knowledge about its anatomical location in fronto-cingulate cortex, and that the fronto-cingulate density landscape of significant spatial attention effects was not flat but contained significant peaks (see Text S1 and Figure S2 for similar results based on an alternative spatial clustering method).

As a second step, we identified anatomical locations on the 2-D cortical flat map that contained a larger proportion of neurons with significant attention effects than expected by probability by performing permutation statistics, which corrected for uneven sampling of neurons across the map (see Materials and Methods for details). We use the term “cluster” to describe adjoined regions of these locations with significant selectivity. Thus, clusters describe statistically significant peaks in the density landscape of attention selectivity. The proportion of neurons with a significant spatial attention effect, as quantified by an ANOVA on the firing rate in the 0.5±0.15 s period, was non-homogeneously distributed within fronto-cingulate cortex, with a significant clustering of effects in LPFC (areas 9 and 6) and ACC (area 24) (Figure 4B). Two smaller clusters of neurons were found in the ventral bank of the principle sulcus (area 46) and in an anterior recording site in area 32 (see contour map in Figure 4B).

Applying the same aforementioned two clustering analysis steps for the contrast between the high-value and the low-value condition revealed that the amount of spatial clustering sharply rose following attention cue onset, first reached statistical significance (*p*≤0.05, *t* test) at 0.3 s, and peaked at 0.4 s after attention cue onset (Figure 4C). Again, this demonstrates that we could reliably predict whether a neuron was value-selective based on its anatomical location in fronto-cingulate cortex. The proportion of neurons with significant target value effects, as quantified by an ANOVA on the firing rate in the 0.5±0.15 s period, following attention cue onset was concentrated within vmPFC (area 32), and extended into area 10, area 9, and posterior towards ACC area 24 (Figure 4D).

### Direction and Heterogeneity of Spatial Attention Effects

**Figure 5:**
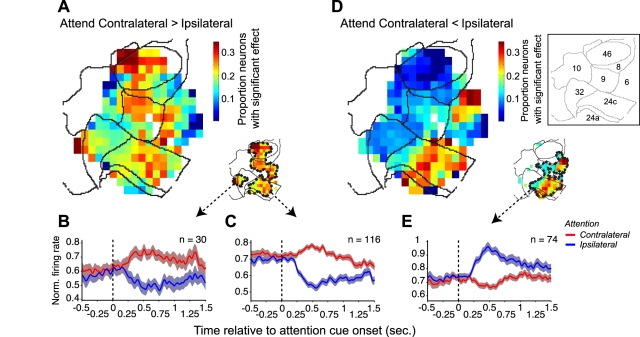
The effect of spatial attention on contra- and ipsilateral targets on the firing rate. (A) Fronto-cingulate distribution of the proportion of neurons that had a significantly (*p*≤0.05) higher firing rate for the contralateral than for the ipsilateral attention condition. The small map on the bottom right shows two separable clusters with a statistically significant spatial concentration of neurons whose firing rates were elevated for the contralateral attention condition. (C) Normalized firing rate as a function of time, separate for the contralateral attention condition (red line) and the ipsilateral attention condition (blue line) for the subset of 30 neurons that were recorded within the smaller spatial cluster within area 32, as indicated by the dashed arrow originating from the small map in (A). Color shading indicates SEM. (B) Same format as (B), but now for 116 neurons that were recorded within the larger contour spanning the complete lateral-to-medial extent of the fronto-cingulate cortex. (D) Same format as (A), but now showing the spatial distribution of the proportion of neurons that had a significantly higher firing rate for the ipsilateral than for the contralateral attention condition. (E) Same format as (B,C), but now for the neurons that were recorded within the contour that spans parts of areas 6, 9, and 24, as indicated by the dashed arrow originating from the small map in (D).

The modulation of neuronal firing rate by spatial attention following attention cue onset could consist of either an enhancement or a suppression of rates for the attend contralateral in comparison to the attend ipsilateral condition. The functional topography for these scenarios varied considerably (Figure 5). Considering the proportion of only those neurons that had significantly higher firing rates at 0.5±0.15 s for the attend contralateral than for the attend ipsilateral condition (*p*≤0.05, *F* test) revealed a widespread distribution of neurons that spanned the complete medial (ACC) to lateral (LPFC) extent of the fronto-cingulate cortex and included a cluster in vmPFC (area 32) (Figure 5A–C). The average firing rate evolution of these neurons with a relative increase in firing rate for the attend contralateral condition in ACC and LPFC reveals a comparatively small increase in firing rates for the attend contralateral condition, and a comparatively strong decrease in firing rates for the attend ipsilateral condition (Figure 5C). Figure S3A,B shows the temporal evolution of the explained variance by the ANOVA (see Materials and Methods for details). The average percent variance of the firing rate modulations explained by the location of the attentional target showed a temporal evolution similar to the firing rates, approaching 7% explained variance for the significantly modulated neurons in this clusters within the first 0.5 s following attention cue onset (Figure S3A,B).

A separate population of neurons had significantly higher firing rates at 0.5±0.15 s for the attend ipsilateral than for the attend contralateral condition, and this population was spatially restricted to the posterior portion of the fronto-cingulate cortex, comprising a single significant cluster in the ACC (area 24) and LPFC areas 6 and 9 (Figure 5D). The average firing rate evolution of these neurons reveals that these neurons predominantly increase their firing rate for the attend ipsilateral condition and only slightly decrease their firing rates for the attend contralateral condition (Figure 5E). The average variance of the firing rate modulations for the significantly modulated neurons in this cluster in vmPFC and ACC explained by the location of the target stimulus approached up to 4% (Figure S3C). Similar to all other contrasts we report, the proportion of explained variance for significantly modulated neurons in the most reliably modulated clusters approached considerably larger values when compared to the variance explained by the total of significantly modulated neurons irrespective of their location in the map, or independent of whether they were significantly modulated or not (see Figure S4).

### Latency and Temporal Evolution of Spatial Attention Effects

**Figure 6:**
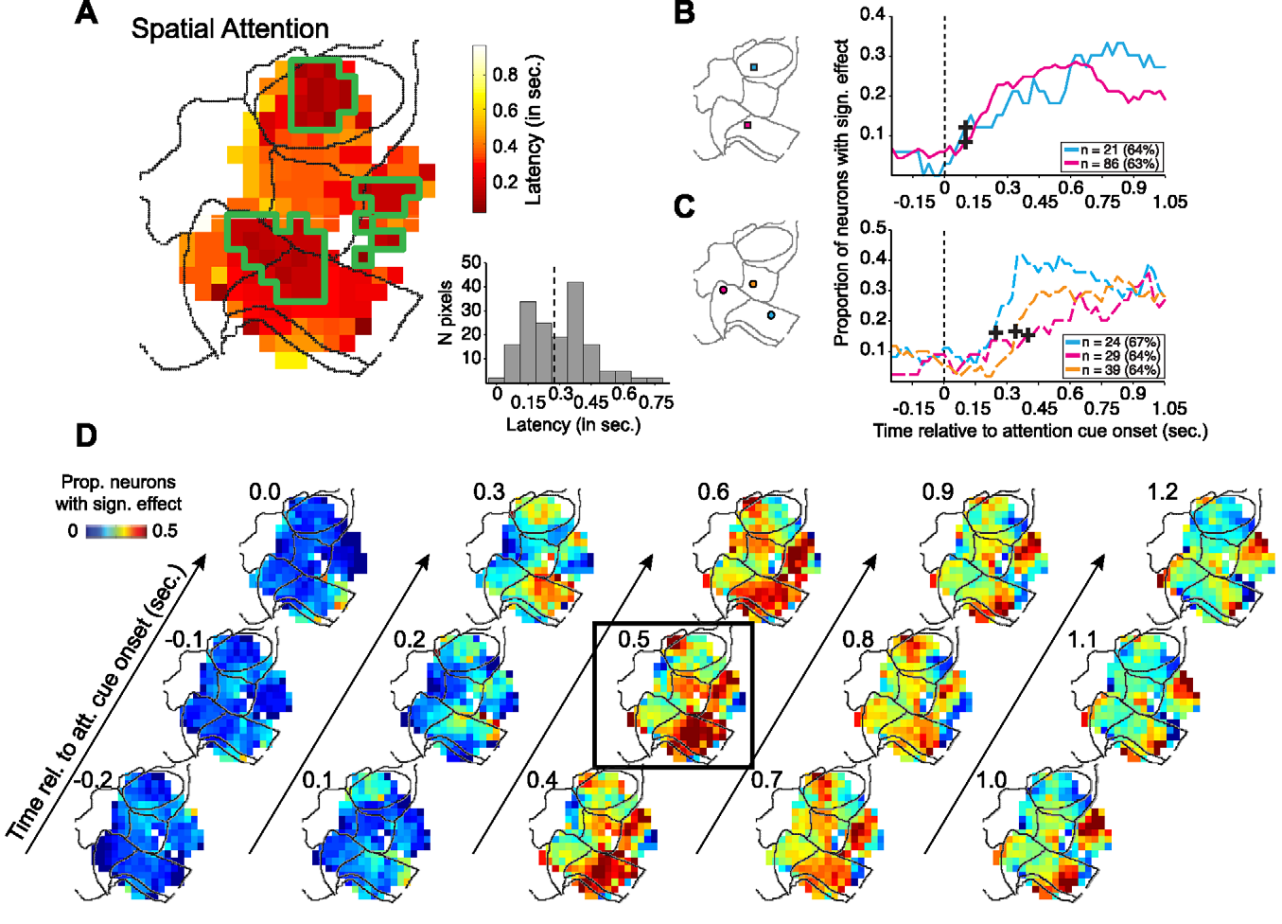
Latency and temporal evolution of spatial attention effects. (A) Fronto-cingulate distribution of latencies of the spatial attention effects. Latency was measured separately for every pixel of the map that had a minimum of five neurons with a significant effect, as the first time after the attention cue onset where for two consecutive time windows the proportion of neurons with a significant spatial attention effect exceeded 3 SD of the proportion of significant effects in the pre-cue period (see text and Figure S5 for details). The panel on the bottom right shows the histogram of latencies across all pixels of the map. (B) Temporal evolution of the proportion of significant spatial attention effects for two pixels in area 46 (*cyan line*) and anterior area 24 (*magenta line*) as indicated by colored squares on the contour map to the left. The cross-hairs highlight the identified latencies. (C) Same format as in (B), showing the temporal evolution of significant spatial attention effects for the three pixels from area 32 (*magenta*), posterior area 24 (*cyan*), and area 9 (*orange*). (D) Fronto-cingulate maps of the proportion of significant spatial attention effects from −0.2 to 1.2 s following attention cue onset.

For each region in the fronto-cingulate map that hosted at least five neurons with a significant spatial attention effect in the 0.5±0.15 s period, we determined the time relative to the attention cue onset at which the proportion of neurons with significant spatial attention effects reached the significance criterion, which was defined as exceeding more than three times the standard deviation of the pre-cue effects (see Materials and Methods and Figure S5 for examples). Under the null hypothesis of no significant spatial attention effects, there is a probability of *p* = 0.006 of crossing the 3 SD threshold at any time-point (one sided *t* test, student *t* distribution with *df* = 6) (see [53]), and the expected distribution of latencies is exponential (Figure S6). Figure 6A shows the spatial topography of latencies for the spatial attention effect, highlighting three clusters in the map with an early onset modulation, and the distribution of latencies across the spatial bins. The distribution of latencies clearly deviates from the exponential one that is expected under the null hypothesis, since it does not have a peak at *t* = 0, and is bimodal with peaks at 0.15 s and 0.3 s. Figure 6B shows the latency and temporal evolution of the proportion of significant spatial attention effects for two sites in ACC (area 24) and in LPFC (area 46), which were located in those regions of the latency map (Figure 6A) with the earliest latencies. Closely adjacent areas in the map showed a slower rise of the proportion of spatial selectivity following cue onset, as illustrated for three examples sites in Figure 6C. To demonstrate the time course of spatial attentional selectivity, Figure 6D illustrates the maps from −0.2 to 1.2 s around the time of attention cue onset in 0.1 s steps. Each map shows the proportion of significant spatial effects calculated for ±0.15 s windows as used in all preceding analysis. The maps show an early rise of spatial attention in area 46, at the intersection of areas 32 and 24, and in area 6, followed by a spread of spatial attention effects across the map, and a subsequent spatial narrowing of attention effects with a sustained focus of attentional modulation in areas 24 and 6.

### Differences in Firing Rates Between the High- and Low-Value Condition

**Figure 7:**
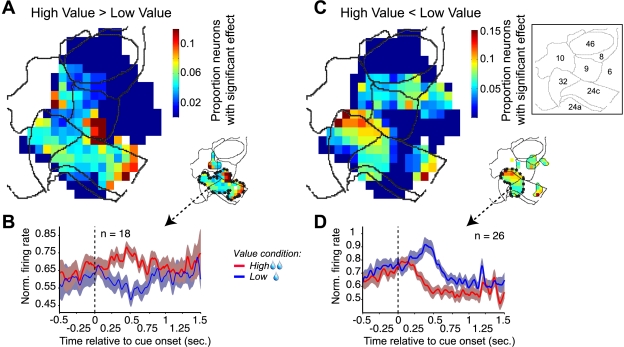
The relationship between firing rate and the association of the cued target's color and liquid volume. (A) Fronto-cingulate distribution of neurons with a significantly (*p*≤0.05) higher firing rate for the high-value than for the low-value condition. The small map on the bottom right shows the cluster with a statistically significant spatial concentration of neurons whose firing rates were elevated when the color of the cued target was associated with a high-value reward. (B) Normalized firing rate as a function of time, separately for the high (red, solid line) and low-value (blue, solid line) conditions, averaged across the 18 neurons recorded within the contour that is shown in the small map in (A), spanning anterior cingulate area 24 and ventromedial area 32. (C) Same format as (A), but showing the spatial distribution of neurons with a higher firing rate for the low-value than for the high-value condition. (D) Same format as (B), but showing the normalized firing rate averaged across the 26 neurons that were recorded within the contour shown in the small map in (C), spanning parts of areas 32 and anterior area 24.

In Figure 4C,D, we show the functional topography of the significant differences in firing rates between the high- and the low-value condition following attention cue onset. Figure 7 illustrates that these effects were based on two partially overlapping neuronal populations with an opposite sign of their value-selectivity, showing higher firing rates for either the high-value condition or for the low-value condition. The first set of neurons was restricted to the medial subsections of fronto-cingulate cortex and spanned areas 32, 24, and most sections of areas 9 and 10 (Figure 7A). These neurons had higher firing rates at 0.5±0.15 s for the high-value condition than for the low-value condition. The average evolution of the firing rate and percent explained variance illustrates a transient attentional effect that leveled off around 0.75 s following cue onset (Figure 7B, Figure S3D). Another set of neurons was located in area 32 and to a lesser proportion in area 8. These neurons showed an average increase in firing rates for the low-value condition and a comparable decrease for the high-value condition (Figure 7C,D). The firing rate modulation was paralleled by an increase in the percent variance explained by the value condition for these neurons that approached 4% (Figure S3E).

### Latency and Temporal Evolution of Value Selective Signals

**Figure 8:**
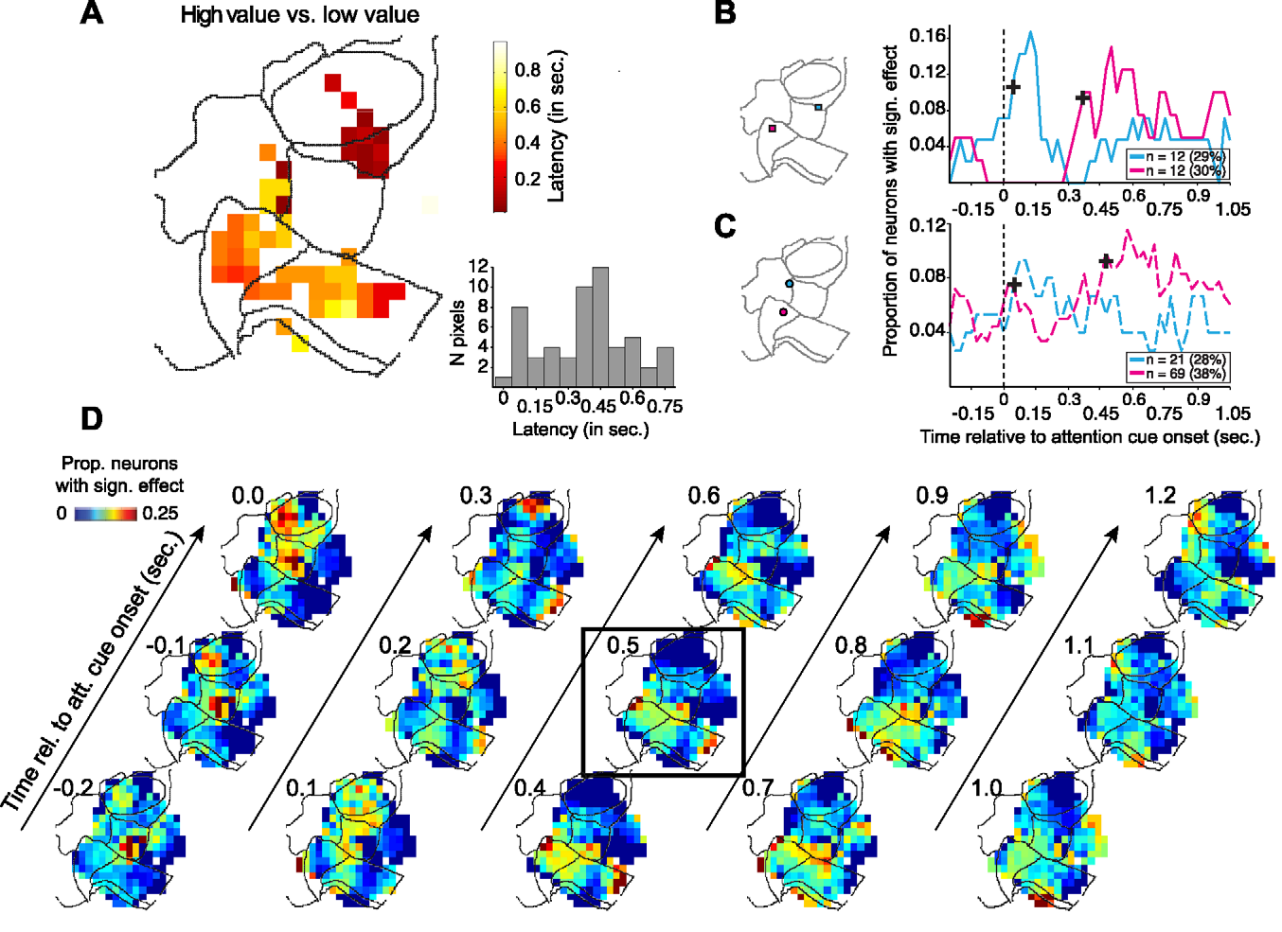
Latency and temporal evolution of the attention effect of target value. (A) Spatial distribution of latencies of the target value effects. Latency was measured separately for every pixel of the map that had a minimum of five neurons with a significant effect, as the first time after the attention cue onset where for two consecutive time windows the proportion of neurons with a significant spatial attention effect exceeded 3 SD of the proportion of significant effects in the pre-cue period (see text and Figure S2 for details). The panel on the bottom right shows the histogram of latencies across all pixels of the map. (B) Temporal evolution of the proportion of significant attention effects of value (high- versus low-value condition) for two pixels in area 32 (*cyan line*) and at the border of areas 8/9 (*magenta line*) as indicated by colored squares on the contour map to the left. The cross-hairs highlight the identified latencies. (C) Same format as in (B), showing the temporal evolution of significant attention effects of value for the two more pixels, one from area 32 (*magenta*) and one from the lateral portion of area 10 (*cyan*). (D) Fronto-cingulate maps of the proportion of significant target value effects from −0.2 to 1.2 s following attention cue onset. The map at 0.5 s reproduces Figure 4D.

We determined the latencies of the value-selective signals at all those sites in the map that contained at least five neurons with significant rate differences between the high- and the low-value condition. Similar to Figure 6, we defined latencies as the times at which the proportion of neurons with significant value-effects reached significance criterion. Figure 8A illustrates the topography of latencies, revealing eight pixel locations with a rapid modulation within 0.1 s following attention cue onset (see histogram in Figure 8A), and a larger number of sites showing a slower onset (peaking around 0.5 s following attention cue onset). The temporal dissociable onset latencies are illustrated for two example sites from areas 8 and 10 that had a rapid onset latency, and for two example sites from area 32 that had a slower, delayed onset of value-selectivity (Figure 8B,C). A more complete picture of the time course of the effect of value condition is shown by the maps in Figure 8D, which were constructed in a similar manner as in Figure 6D. Taken together, these maps show that value-selectivity was already present in areas 46 and 9 at the time of attention cue onset, but transiently increased thereafter at separable nearby pixels of the same areas. In contrast, target value selectivity in areas 32 (and within area 24) rose with a longer latency.

### Integration of Selective Spatial and Reward Expectancy Information

**Figure 9:**
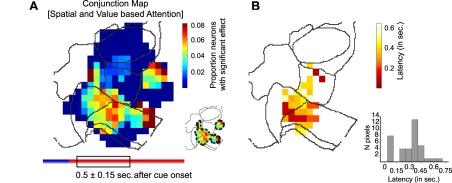
Conjunction map of spatial attention and target value effects. (A) Spatial distribution of neurons showing a significant main effect of spatial attention (contra- versus ipsilateral attention) *and* expected outcome value (high- versus low-value condition). The map is colored at all pixels with more than 10 recorded neurons, revealing that combined selectivity for location and value of attentional targets was restricted to two clusters within fronto-cingulate cortex (shown in the small contour map on the right bottom). (B) Spatial distribution of latencies of combined effects of spatial attention and target value (in same format as in Figures 6A and 8A). Latency was measured per pixel of the map and only if there were more than five neurons with a significant conjunction effect for a pixel. The panel on the bottom right shows the histogram of latencies across all pixels of the map.

So far, our analyses considered spatial attention selectivity and value-selectivity separately. To identify neurons that signaled both attentional dimensions following attention cue onset, we computed a “conjunction map” that shows the topography of the proportion of neurons whose firing rates between 0.5±0.15 s were significantly modulated by both the cued target location (*p*≤0.05, *F* test; contra- versus ipsilateral attention) and value condition (*p*≤0.05, *F* test; high value versus low value) (Figure 9A). This conjunction map shows that both attentional dimensions were signaled by a group of neurons within vmPFC (area 32) and ACC (area 24), with a larger cluster of neurons located at the intersection of area 32/24. A second group of neurons in area 6, posterior to area 8, was also selectively modulated by both target location and value. The latency analysis of the combined encoding, restricted to grid locations with at least five significant conjunction-coding neurons, shows that the conjunction of spatial attention selectivity and value-selectivity at the intersection of areas 32 and 24 and at two neighboring sites in area 9 occurred with a rapid onset latency (Figure 9B).

### Topography of the Effects of “Stimulus Value” and the Interaction of Spatial Attention and Value Selectivity

**Figure 10:**
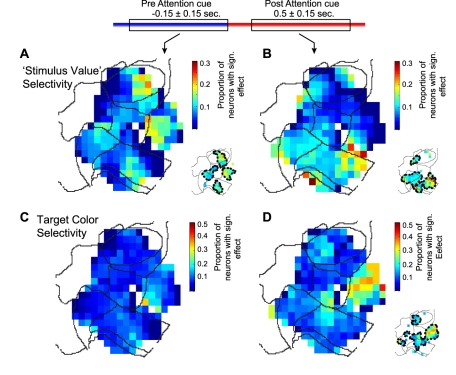
Spatial distribution of “stimulus value” effects and feature-based attentional effects. (A) Spatial distribution of neurons showing a significant main effect for “stimulus value”, which contrasts the value and spatial effects independent of whether a stimulus is the target for covert attention, i.e. contrasting “contralateral-high value” versus “contralateral-low value” conditions (see text for details). (B) The small panel to the bottom right in (B) illustrates scattered clusters with a statistical sign. proportion of neurons whose responses are modulated by the color of the attentional target (*feature-based* attentional effects). (C,D) Same format as in (A,B), but for neurons with a significant effect for the color of the stimuli (red versus green color) before (C) and after (D) attention cue onset. The small maps to the bottom right in each panel illustrate scattered clusters with a statistical sign. Proportion of neurons whose responses are modulated by the color of the attentional target (*feature-based* attentional effects).

To examine the interaction between target value and target location, we contrasted trials where the contralaterally presented grating was associated with a high-value outcome and trials where it was associated with a low-value outcome. Before the attention cue onset (when no spatial attention condition can be defined yet), this contrast measures the spatial specificity of value selectivity (which we call “stimulus value”), irrespective of whether the stimulus will later be attended. Figure 10A shows the topography of the proportion of significant stimulus value effects before attention cue onset (for −0.3 to 0 s), revealing four spatial clusters of stimulus value coding neurons scattered across LPFC (area 46 and area 6), vmPFC (area 32/10), and ACC (area 24).

After the attention cue onset, this contrast accounts for the interaction between the spatial attention condition and the value condition, since it attains a value of 1 both for the combination of the attention contralateral with the high-value condition and the attention ipsilateral with the low-value condition. The spatial clustering of the interaction of spatial selectivity and value selectivity following attention cue onset revealed a cluster spanning vmPFC and ACC (Figure 10C).

### Topography of Color Selectivity

In addition to the spatial location, the color of the attentional target stimulus was an additional feature dimension that needed to be encoded in order to shift attention by correctly applying the association rule between the fixation cue color and the grating stimulus color. To test for feature selectivity, we contrasted “attend green” and “attend red” conditions with the multifactorial ANOVA (see Materials and Methods) in 0.15 s windows before and after attention cue onset. Figure 10C,D shows the topography of significant feature selectivity for the 0.5±0.15 s period, illustrating several smaller clusters across the map, and one larger cluster spanning areas 6, 9, and dorsal ACC (area 24c) that conveyed color-selective information about the cued target stimulus.

## Discussion

Our results identified spatially confined clusters of neurons within subdivisions of the fronto-cingulate cortex of primates that contained specific information about the attentional targets around the time of covert attentional stimulus selection. Our task isolated information about the value associated with an attentional target from the attentional targets' location. Both of these variables were manipulated independently from the direction of the overt saccadic choice on the rotation direction of the target stimulus, ruling out possible influences from spatially specific motor intentions and action related valuation processes [54]–[58]. The behavioral analysis revealed that, within a narrow time window of 0.4 s following the attention cue onset, behavioral accuracy was higher if the cued target stimulus was associated with a higher reward outcome (Figure 1E), consistent with previous findings that top-down attention can be facilitated (impaired) when the target is of higher (lower) valence than distracting stimuli [4],[6]–[10].

### Valuation Processes Within PFC During Shifts of Attention

We found that this behavioral signature was paralleled by value-selective response modulation of neurons located within vmPFC (areas 10, 32), ACC (area 24), and LPFC area 8. These behavioral and neuronal findings illustrate that stimulus valuation processes are recruited during covert shifts of attention and are represented in the macaque brain independently of valuation processes pertaining to actions and motor plans [12],[59],[60]. This finding corroborates computational frameworks that rely on independent coding of stimulus and action related values, based on the acknowledgment that covert stimulus selection typically precedes overt action selection [12].

Value selective signals were spatially dissociable from the anatomical clustering of the spatial attention signals that were based on top-down goal/rule information (Figures 4, 5, and 7). The largest proportions of value-selective neurons were found within vmPFC (areas 10, 32) (Figure 7). This finding is consistent with the recent hypothesis that neuronal circuitry within the larger vmPFC, including orbitofrontal subdivisions (see Averbeck and Seo, 2008), encodes a value map, that is recruited to inform overt choice behavior and decision making [17],[61],[62]. This suggestion is corroborated by an increasing number of single neuron macaque and rodent studies, as well as human fMRI studies, that identify areas within the larger vmPFC that encode valuation signals pertaining to simple stimuli, complex objects, “goods,” and abstract monetary values [11],[17],[18],[20],[61],[63]–[70].

One notable extension to the vmPFC-based view was a reliably observed cluster of neurons within LPFC area 8 that increased spiking activity with a rapid onset after the attention cue if attention was directed to the target stimulus that was associated with a low outcome value (Figure 7). The behavioral analysis (Figure 1E) suggests that shifting attention to these “lower incentive” stimuli requires the system to overcome a motivational bias of attending higher incentive stimuli [4]. Such an overruling of positive incentive values has been conceptualized as “self-control” processes in human studies, and consistent with our findings in the macaque, is associated with activation of the dorsolateral PFC, in addition to rostral ACC in humans [71].

### Integration of Valuation Processes During Attentional Control

We found a conjunctive presence of value and spatial attentional selectivities in the same neurons only at the intersection of vmPFC and ACC (Figure 9). Furthermore, interactions between attention selectivity and value selectivity were predominantly found in a cluster that spanned areas 24 and 32 (Figure 10C). A subset of these neurons had an early-onset latency of selective response modulation and could thereby contribute to initiating the shift of attention (Figure 6) [72]. Anatomical connectivity profiles of both areas are distinct: Area 24 pertains to a dorsal “prefrontal” subdivision including area 9 and is connected predominantly with premotor structures, while area 32 constitutes part of the ventromedial frontal subdivision with strong connections to orbitofrontal areas and “limbic” structures (including amygdala, ventral striatum, and the hippocampal formation) [73]–[75]. These dissociable connectivity profiles render the intersection of rostral area 24 and area 32 an ideal integration zone for cognitive-related, sensory-motor information (such as the location of task relevant stimuli, or the color-location association underlying the shift of attention), on the one hand, with “more” reward-related information (such as stimulus-value associations), on the other hand [76]–[80].

### Spatial Attention in Dorsal and Ventral Lateral PFC

We found spatial attention selectivity to be distributed across the complete medial-to-lateral extent of the fronto-cingulate cortex, spanning areas 24, 6, 8, 9, and 46 (Figure 5). The anatomical clustering of these spatial attention signals was largely spatially dissociable from value signals (Figures 4, 5, and 7). The early onset of attention signals in LPFC (Figure 6) suggests that these neuronal groups could contribute to the initiation of the shift of attention, thereby constituting one source of the top-down attentional biasing signals [80],[81]. The most posterior neuronal group with early onset signals was located in area 6, which has also been labeled dorsolateral area 8 in previous studies, and is anatomically closest to the fundus of the arcuate sulcus (containing the FEF), which hosts neurons with spatially confined receptive fields and rapid onset target selection signals [31],[49],[82]–[86]. The rapid emergence of spatial selectivity following the attentional cue is consistent with results from previous studies deploying delayed matching tasks, visual search tasks, or spatial attention tasks [35],[39],[85],[87]–[89].

A second “early-onset” cluster was located within the anterior aspect of area 46 and spanned the ventral and dorsal bank of the principal sulcus. Following previous suggestions, this ventrolateral portion of the PFC may serve as a critical sensory gateway into prefrontal cortex, integrating feature and spatial information of task relevant, attentional target stimuli [29],[44]. Our electrophysiological findings strongly support the conclusions from a previous lesion experiment: bilateral ablation of the ventral LPFC in macaque monkeys impairs the attentional selection of relevant stimuli as soon as there is a spatial separation of the sensory target stimulus from the site of the required action that leads to reward, i.e. as soon as task demands require attentional stimulus selection, rather than intentional action selection [29].

### Spatial Attention Signals in ACC

Our findings also suggest and specify the role of the ACC in the control of interference from distractors during selective attentional processing. Our results dissociate the functional association and anatomical site of the discussed rostral, anterior portion of area 24 (bordering vmPFC) from the more caudal and posterior area 24. This posterior portion of the ACC has been the recording site in many previous electrophysiological studies of the ACC, being located well anterior to the rostral cingulate motor area [54],[56],[90]–[97]. We showed that this posterior subregion contains neurons that develop selective attentional response modulation only gradually within the first 0.5 s after attention cue onset (Figures 5 and 6). This gradually evolving spatial selectivity in area 24 was unique because it reflected the largest proportion of neurons with spatial selectivity across the fronto-cingulate map (Figure 4), showed the most heterogenous response modulations (with about equal numbers of neurons increasing and decreasing their activity with contralateral shifts of attention) (Figure 5), and maintained spatial selectivity beyond the immediate attentional shift period (Figure 6 and Figure S3A–C).

These functional signatures of ACC neurons suggests a pivotal role for them in sustaining selective attention on one among many available and possibly distracting (“conflicting”) stimuli. We propose that the most parsimonious concept to account for these selective response modulations is the “control of interference” [40], which is consistent with the proposed key role of dorsal ACC in humans to gate salient, sensory afferents from influencing attentional top-down control signals [44]. According to this gating hypothesis, neurons in ACC inhibit neuronal activity in visual and parietal cortex that conveys information about physically salient distractors. In our task, distractor and target stimuli had identical physical salience, thus requiring the proposed gating mechanism to prevent the distractor from influencing attentional prioritized processing of the target stimulus.

The functional consequences of neuronal activity in ACC that we described as “sensory gating” and “interference control” could likewise be described under the functional term “conflict monitoring” [43]. “Conflict monitoring” processes have the particular connotation of playing an active role to resolve conflict whenever it becomes more prevalent. It will require future studies that manipulate more explicitly the degree of sensory interference during attentional processing to determine whether neurons in ACC contribute actively to resolve conflicting and interfering sensory information.

In summary, our data provide evidence that valuation processes conveying stimulus-specific reward expectancies are recruited during covert attentional stimulus selection [5],[11]. These valuation processes integrate with top-down attentional control information within confined clusters in fronto-cingulate cortex following time courses that allow us to bias the initiation of attentional shifts and to control sustained selection beyond the immediate attentional shifting period.

## Materials and Methods

### Procedures

We collected data in two male macaque monkeys following guidelines of the Canadian Council of Animal Care policy on the use of laboratory animals and of the University of Western Ontario Council on Animal Care. Extra-cellular recordings commenced with 1–6 tungsten electrodes (impedance 1.2–2.2 MΩ, FHC, Bowdoinham, ME) through standard recording chambers (19 mm inner diameter) implanted over the left hemisphere in both monkeys. For monkey R, we initially recorded approximately 30 sites in the right hemisphere through an additional chamber implanted with an oblique angle over the midline. This chamber allowed a perpendicular penetration of the principal sulcus, but at the risk of penetrating the dura at an extreme angle and close to major blood vessels, which prevented further usage. For monkey M, we re-positioned the recording chamber once in order to allow access to more anterior aspects of the prefrontal cortex and cingulate sulcus, and to align recordings to the same anterior-to-posterior extent of the frontal cortex as covered with recordings obtained in monkey R (see below: Reconstruction of Recording Sites). Electrodes were lowered through guide tubes with software controlled precision microdrives (NAN Instruments Ltd., Israel) on a daily basis, through a recording grid with 1 mm inter-hole spacing. Before recordings began, anatomical 7T MRIs were obtained from both monkeys with ear channels made visible with vitamin E for later horizontal alignment, and with visualization of possible electrode trajectories in the recording grid using iodine (see Figure 2A,B).

Data amplification, filtering, and acquisition were done with a multi-channel processor (Map System, Plexon, Inc.), using headstages with unit gain. Spiking activity was obtained following a 100–8,000 Hz passband filter and further amplification and digitization at 40 kHz sampling rate. During recording, the spike threshold was always adjusted such that there was a low proportion of multiunit activity visible against which we could separate single neuron action potentials in a 0.85 to 1.1 ms time window. Sorting and isolation of single unit activity was performed offline with Plexon Offline Sorter (Plexon Inc., Dallas, TX), based on principal component analysis of the spike waveforms, and strictly limiting unit isolation to periods with temporal stability.

Experiments were performed in a sound attenuating isolation chamber (Crist Instrument Co., Inc.). Monkeys sat in a custom-made primate chair viewing visual stimuli on a computer monitor (85 Hz refresh rate, distance of 58 cm). The monitor covered 36°×27° of visual angle at a resolution of 28.5 pixel/deg. Eye positions were monitored using a video-based eye-tracking system (ISCAN, Woburn, USA, sampling rate: 120 Hz) calibrated prior to each experiment to a 5 point fixation pattern (one central fixation point and the remaining four points offset by vertical 8.8° and horizontal 6° toward the four corners of the monitor). Eye fixation was controlled within a 1.4–2.0 degree radius window. During the experiments, stimulus presentation, monitored eye positions, and reward delivery were controlled via MonkeyLogic (open-source software http://www.monkeylogic.net) running on a Pentium III PC [98],[99]. Liquid reward was delivered by a custom-made, air-compression controlled, mechanical valve system with a noise level during valve openings of 17 dB within the isolation chamber.

### Behavioral Paradigm

Monkeys performed a selective attention task requiring a two-alternative forced-choice discrimination on the rotation direction of the attended stimulus, and ignoring rotations of the distracting stimulus presented in the other visual hemifield (Figure 1D). The task is a modification of a previously used selective attention task [49]–[51]. Monkeys initiated trials by directing and maintaining their gaze on a centrally presented, grey fixation point (on a black (0.6 candela) background). After 0.3 s, two black/white grating stimuli appeared drifting within two separate apertures, and their respective colors were changed to either black/red (max. 31 candela) or black/green (max. 40 candela) another 0.4 s later. Within 0.05 to 0.75 s after this change in grating color, the color of the central fixation point changed to either red or green, which cued the monkeys to covertly shift attention towards the location where the color of the grating matched the color of the fixation point. In order to obtain a liquid reward, the monkeys had to discriminate a smooth, transient clockwise from a counterclockwise rotation (see Stimuli for details) of the cued target grating by making respectively up- and downward saccades towards one out of two response target dots. This rotation of the cued target grating occurred at random times within 0.05–4 s drawn from a uniform (flat) probability distribution. The angle of rotation was adjusted during training to ensure ≥85% of overall correct responses to the grating.

To infer selective attention to the cued target stimulus, in half of the trials the distractor, i.e. the grating whose color did not match the color of the fixation point, rotated before the target. The distractor change times were likewise drawn from a uniform probability distribution. The uniform distribution of target and distractor change times caused exponentially rising hazard rates for target and distractor change times, which did not differ for “contra-“ and “ipsilateral,” or “high-value” and “low-value” attention conditions. In every trial, we chose the location, color, and rotation direction (and thereby saccadic response direction) of target stimuli randomly and independently from another according to a Bernoulli distribution.

A trial was considered correct and was followed by liquid reward if the monkeys made a saccade to the correct one of the two peripheral response dot targets, which had a fixed correspondence to the rotation direction of the target stimulus, and were presented at, respectively, 5 degrees up and down relative to the fixation point. Correct saccadic responses had to occur within 0.05 to 0.75 s following rotation onset, and saccadic fixation breaks outside of this time window were considered errors, as were failures to respond to the target rotation. For all analyses, only error trials were considered where fixation was broken *after* a stimulus rotation onset, i.e. either after the onset of the distractor change when it changed before the target or after the onset of the target change.

The volume of the liquid reward for correct responses was dependent on the stimulus color, with red and green associated with 0.76 and 0.4 ml. Color-reward associations were changed every 30 correctly performed trials with identical numbers of trials with red and green attentional targets. These alternating blocks were interleaved by five fixation trials, where the monkey received a 0.3 ml volume reward for keeping fixation on a yellow fixation point until it changed to blue, which triggered liquid delivery. These fixation trials had the same peripheral visual stimulation and timing parameters than the attention trials.

### Stimuli

Stimuli were square wave gratings with “rounded off” edges (Figure 1D), moving within a circular aperture at 1.0 degrees per second, a spatial frequency of 1.4 cycles per degrees, and a radius of 1.5–2.2 degrees. Gratings were presented at 4.2 degrees eccentricity to the left and right of fixation. The grating on the left (right) side always moved within the aperture upwards at −45 (+45) degrees relative to vertical. The angle of rotation that was adjusted during training to ensure ≥85% of overall correct responses to the grating (see Behavioral Paradigm) ranged between ±13 and ±19 degrees. The rotation proceeded smoothly from the standard direction of motion towards maximum tilt within 60 ms, staying at maximum tilt for 235 ms, rotated back to the standard direction within 60 ms, and continued moving at their pre-change direction of motion at −45 or +45 degrees relative to vertical thereafter.

### Reconstruction of Recording Sites

The anatomical site of each recorded neuron was reconstructed and projected onto the 2-D flat map representation of a standardized macaque brain (F99) available within the MR software *Caret* (Figure 2) [52]. Reconstruction began by projecting each electrode's trajectory onto the 2-D brain slices obtained from 7T anatomical MRI images, using the open-source OsiriX Imaging software [100] and custom-written MATLAB programs (Mathworks Inc.), utilizing the iodine visualized electrode trajectory within the electrode grid placed within the recording chamber during MR scanning. We drew the coronal outline of the cortical folding of the MR grey scale image to ease the comparison of the individual's monkey brain slices to standard anatomical atlases, before projecting the electrode tip position into the standardized macaque brain (“F99”) available in Caret [52]. Note that we initially reproduced the individual monkey brains within the Caret software to validate similarity and derive the scaling factors to match the lower resolution monkey MRs to the higher resolution standard F99 brain. We then manually projected, under visual guidance, the electrode position to the matched location in the standard brain in Caret [101].

After identifying all recording sites within the standard brain, we used the Caret software to render the brain to a 3-D volume, spherically inflated and cut it to unfold the brain into 2-D space (see Figure 2). In an independent procedure we visualized major anatomical subdivision schemes of the fronto-cingulate cortex, using the scheme from Barbas and Zikopoulus (2007) [77] as a major reference throughout the manuscript. We visualized two alternative subdivision schemes with their anatomical labels in Figure S1.

We subjectively estimate that the complete procedure from documenting precisely the recording depth, identification of the recording location in the monkeys MR slice, to the placement of the electrode position in the standard macaque brain introduces a potential maximal error of 3 mm. The more common, and still unsystematic, error will be within 1 mm range. Anatomical reconstruction was conducted entirely independent of (and blind to) the functional analysis of the neuronal data and their projection onto the anatomical 2-D map.

### Data Analysis

Analysis was performed with custom MATLAB code (Mathworks, Natick, MA), utilizing functionality from the open-source fieldtrip toolbox (http://www.ru.nl/fcdonders/fieldtrip/). Analysis of spiking activity was based on convolving spiketrains of individual trials with a gaussian (SD 30 ms). The resulting spike density functions were aligned in time to the onset of the attentional cue. To prevent any influence from transient stimulus changes on cue-aligned analysis, we removed time epochs at which the color onset was within 0.3 s before cue onset, and limited analysis to the time of any stimulus change after cue onset, which could be the rotation either of the target or of the distractor. We further limited analysis to neurons with >1 Hz average firing rate during the cue period, and a minimum of 30 trials per attention condition.

### ANOVA Analysis

To analyze whether neuronal spiking activity was modulated by attention (“attend contra- versus ipsilateral,” and “high-value” versus “low-value” condition), we performed multifactorial, first-order ANOVAs of four explanatory variables, namely spatial attention condition, value condition, cued target color, and “stimulus value.” Stimulus value attains a value of 1 for the combination of attention contralateral and high-value condition or the combination of attention ipsilateral and the low-value condition, and 0 for the other combinations. It thereby does not represent a main effect, but represents the interaction term of Spatial Attention Condition×Value Condition. Interactions of attention and value with color were not analyzed.

For a time-resolved analysis of selectivity for the four explanatory variables, ANOVAs were applied for ±0.15 s time windows stepped every 0.05 s around the time of the attention cue onset (from −0.25 s before to 1.5 s after the attention cue onset) to identify whether neurons were significantly (*p*≤0.05, *F* test) conveying selective information. Results obtained by using ROC analysis (see Figure 3C,D) with permutation statistics to derive significance provided similar results to those obtained from ANOVA, but are not shown.

To provide a measure of the effect sizes we calculated the percent of explained variance for the four explanatory variables by means of simple-effect ANOVAs for the same time windows as above. We calculated the variance component of the explanatory factor (σ^2^a) relative to the total variance (σ^2^) as: 100*(σ^2^a/(σ^2^a+σ^2^)) (see, e.g., [102]).

### Mutual Information Analysis

A mutual information analysis was used to test for each time epoch from −0.25 up to 1.5 s after attention cue onset, whether neurons showing significant attentional or value modulation were more likely recorded at similar locations on the flat map compared to the null hypothesis of a random spatial distribution of significant effects. For every neuron, we determined the statistical significance of the attention or value-selectivity, which was captured by a binary variable *S* (i.e., 0 or 1), and its location on the map. A neuron's location on the map was described by the random variable *L*, which took one out of *N* values (the *N* bin numbers), using the same bins as in Figure 2D. We then estimated the (Shannon) mutual information between statistical significance and location. Mutual information is defined as the difference between unconditional (for the given analysis, ignoring attention or value condition) and conditional (for the given analysis, conditional on attention or value condition) entropy (a measure of the uncertainty about a random variable). In our case, the mutual information was defined as I(*S*;*L*) = H(*S*)−H(*S*|*L*), where H(*S*) was the unconditional entropy of *S*, and H(*S*|*L*) the conditional entropy of *S* conditional on *L*. Thus, mutual information is defined as a reduction in uncertainty (measured by entropy), estimated using the bins in Figure 2D, about the random variable *S* (significance) by observing the random variable *L* (location). Mutual information quantifies how well a decoder can predict the statistical significance of a neuron given knowledge of its location. To control for the well-known fact that mutual information is a quantity that can be positively biased by sample size (e.g., see [103]), we performed a shuffling procedure (*N* = 1,000) by randomly interchanging the locations of the neurons, keeping the total number of neurons at each bin constant. We tested for statistical significance by determining if the mutual information exceeded 1.64 standard deviations of the randomization distribution of the mutual information, corresponding to a one-sided test with *p*≤0.05. While the mutual information estimator can be (but not necessarily) positively biased by sample size, discretizing the response space (location) leads to a loss in information relative to the differential (i.e., continuous) mutual information whose estimate we seek.

In addition, we also tested for spatial concentration of attention and value effects by performing a nearest neighbor analysis (see Text S1 and Figure S2). There exists a close relationship between the nearest neighbor analysis and the mutual information analysis. A well-known binless estimator of the entropy of a continuous, N-d random variable is based on nearest neighbor distances [104],[105]. A spatial distribution with a low entropy corresponds to small nearest neighbor distances, and a peaked density landscape. Intuitively, this can be understood from the fact that there are many points at the density peaks, and that these points have small nearest neighbor distances (although a strict mathematical relationship between entropy and nearest neighbor distance exists; see [104]). A spatial distribution with a high entropy corresponds to large nearest neighbor distances and a more uniform density landscape.

### Identification of Clusters

To identify anatomical locations on the flat map that contained a larger proportion of neurons with significant attention effects than expected by probability, we performed permutation statistics, which corrected for uneven sampling of neurons across the map. To test against the null hypothesis that there is a homogenous distribution of the proportion of significant effects across the map, we first calculated the proportion of significant neurons within 4 mm circular radius around the intersections of a regular grid overlaid onto the 2-D flat map representation of the fronto-cingulate cortex (using 3 mm or 5 mm radii resulted in qualitatively similar results). We used a 2 mm spacing to obtain a smooth and homogenous surface across the map. We then obtained for each intersection a random distribution of the proportion of significant neurons after randomly assigning the significance of the neuronal population to recording locations, which kept the number of neurons at each intersection constant. We limited the analysis to only those map intersections with at least 10 recorded neurons. Statistical significance was identified if the observed proportion of significant neurons at an intersection exceeded the [mean * 1.96 the standard deviations] of the random distribution, corresponding to a one-tailed test with *p*≤0.01.

### Latency Analysis of Neuronal Selectivity

To quantify the latency of attentional information for each intersection, we calculated the proportion of neurons with a significant effect at successive 0.05 s time intervals around the time of the attention cue onset. For each neuron, we then identified the variability (i.e., the standard deviation) of the proportion of significant neurons before the cue onset (across six time points from −0.25 to 0 s) and determined the latency of attention as the first of two consecutive time bins after cue onset, when the proportion of neurons at this intersection exceeded the [mean * 3 standard deviations] of the pre-cue effects. This latency measure was found to reliably capture the time of rise in the proportion of neurons for all intersections as illustrated for several examples in Figure S5 and has been validated in previous studies (e.g., [53]).

## Supporting Information

Figure S1 Three anatomical schemes subdividing the fronto-cingulate cortex into areas according to difference in cytoarchitecture and identified afferent and efferent connectivity. (A) Subdivisions proposed by Barbas and Zikopoulus (2007) [77], entered as colored shadings into the standard F99 macaque brain available in Caret rendered in 3-D (*top panel*), semi-inflated 3-D volume (*middle panel*), and flattened into a 2-D map representation using the Caret software package [52]. (B and C) Same format as in A but with area subdivisions proposed by Petrides and Pandya (2007) [83] (B) and by Saleem, Kondo, and Price (2008) [82] (C). Note the overall agreement across subdivision schemes from different labs (see Supporting Information for more details).
(EPS)

Figure S2 Spatial topography of the effects of spatial attention and target value during the attention shift. (A) Spatial clustering coefficients, based on nearest neighbor analysis, for a significant (*p*≤0.05) main effect of spatial attention (attend contra- versus ipsilateral stimulus) as a function of the time relative to attention cue onset. The grey shading denotes time epochs with significant spatial clustering. (B) Same format as (A), but showing the clustering coefficients and spatial distribution of the proportion of neurons with significant effect for target value (attention to target with high versus low expected outcome).
(EPS)

Figure S3 Temporal evolution of explained variance (EV) by spatial attention and value selectivity. (A) Average percent variance explained (*x*-axis) around the time of the attention cue onset (*y*-axis) by spatial attention for those neurons in vmPFC (area 32) with a sign. Enhanced firing rate for attention shifts to the contralateral versus ipsilateral target stimulus (see topographic outline to the right and Figure 5B of the main text). (B) Same as in (A) but for the set of neurons that were recorded within the larger contour spanning the complete lateral-to-medial extent of the fronto-cingulate cortex (see topographic outline to the right and Figure 5C of the main text). (C) Same format as in (A,B), but for the subset of neurons that showed significantly enhanced firing rate when attention shifted to an ipsi- versus contralateral target (see map to the right and Figure 5E of the main text). (D,E) Same format as in (A–C), but showing the percent explained variance for neurons with significantly enhanced (D) or significantly reduced (E) rate when attention shifted to the target with higher reward outcome expectancy (see Figure 7B and Figure 7D). The grey shading indicates SEM.
(EPS)

Figure S4 Average percent explained variance for the set of neurons with main effects and in total. (A) Box plots (showing median, 25th and 75th percentiles within limits of the box and the range) of the average percent variance explained (EV) by the spatial attention condition (contra- versus ipsilateral) and the value condition (high versus low value associated with the attended target stimulus). The average EV is based on neurons with a significant main effect. The smaller boxplot to the right shows the average EV across all recorded neurons irrespective of the single cell significance. (B) Same format as in (A), but showing the average percent EV for the effects of “stimulus value” (the interaction of stimulus value and stimulus location *before* the attention cue onset), the interaction of spatial attention and target value, and the main effect of the target color (attend red versus green).
(EPS)

Figure S5 Examples of the latency analysis for spatial attention effect. The anatomical locations of example sites are indicated as colored symbols in the flat map outline on the top left. The individual panels show for each of the example sites (a single pixel in the flat map) the temporal evolution of the proportion of significant neurons. The color symbols in the top left corner of the panels match to the symbol in the flat map. The dashed horizontal line denotes threshold (three SD beyond pre-cue average), which was the criterion for identification of the latency of the attentional effect for each pixel on the map, Two successive threshold crossings were required to be identified as latency, which is illustrated as a red vertical line in each panel.
(EPS)

Figure S6 Exponential distribution of latencies that is expected under the null hypothesis. Under the null hypothesis of no significant attention effects in the post-cue period, there is a certain probability *p* of crossing the 3 SD (Standard Deviation) threshold. The waiting time until the first trigger after the cue onset follows an exponential distribution. The probability of crossing the 3 SD threshold by a random trigger in the first bin equals *p*_1_ = *p*, and the probability of crossing it in the *n*th bin equals *p*_n_ = p*(1−p)^(n−1)^. Shown is *p_n_* as a function of the bin number, for *p* = 0.006 (probability of 3 SD crossing according to a one-sided *t* test with *df* = 6).
(EPS)

Text S1 Time to shift attention.
(PDF)

## Abbreviations

ACC: anterior cingulate cortex
LPFC: lateral prefrontal cortex
vmPFC: ventromedial prefrontal cortex
