# Supplementary material for: Specific Contributions of Ventromedial, Anterior Cingulate, and Lateral Prefrontal Cortex for Attentional Selection and Stimulus Valuation
Source: PLoS Biol. 2011 Dec 27;9(12):e1001224. doi: 10.1371/journal.pbio.1001224 (PMC3246452; doi:10.1371/journal.pbio.1001224)
Supplement: Text S1 — Time to shift attention. (PDF) [file pbio.1001224.s007.pdf]

Supporting Information for:

Specific contributions of ventromedial frontal, anterior cingulate and lateral prefrontal cortex for attentional selection and stimulus valuation

Daniel Kaping[1], Martin Vinck[2], Iman Janemi[1], Michelle Bale[1], R. Matthew Hutchison[1], Stefan Everling[1,3] & Thilo Womelsdorf[1]

[1] *Department of Physiology and Pharmacology, University of Western Ontario, 100 Perth Drive, London, Ontario N6A 5K8,Canada*
[2] *Cognitive and Systems Neuroscience Group, Center for Neuroscience, University of Amsterdam, Amsterdam, the Netherlands*
[3] *Robarts Research Institute, N6A 5K8 London, Ontario, 100 Perth Drive, Canada*

Supplementary Information SI 1.

Time to shift attention. We evaluated the time for the monkeys to shift attention following cue onset by analyzing behavioral accuracy and reaction time as a function of the time between cue onset and the stimulus change (Figure 1 E,F). We calculated the proportion of correct trials and saccadic reaction times to the change within ±0.125 sec. wide sliding windows every 0.05 sec. following cue onset. With regard to accuracy, monkeys reached on average a plateau discrimination performance for stimulus changes occurring as early as 0.2 sec. following stimulus change. However, accuracy was significantly better ($p <= 0.05$) when cued to the high-value target compared to the low-value target during the attentional shift period from 0.15 to 0.4 sec. following cue onset (see *main text* and Figure 1 E). Compared to accuracy, saccadic reaction times reached a consistent plateau level far later relative to cue onset (Figure 1 E, *bottom panel*). To quantify the duration monkeys required to reach asymptotic reaction times, we compared the

saccadic reaction times across the early time windows to the reaction time average of the later time windows (1.5 sec. to 4 sec. following cue onset). Unexpectedly, reaction times were significantly slower than the plateau level (mean: 0.279 sec.) for all time windows before 0.8 sec. ($p <= 0.05$, *unpaired ttest*) and reached plateau reaction times only thereafter (black arrow in Fig. 1E, bottom panel). This result suggests that shifts of the attentional focus to the cued target stimulus was fully established on average only at 0.8 sec. following cue onset. These findings indicate that shifting attention is a gradual process of attentional engagement of 'processing resources': Above-chance, but slower than average performance for stimulus changes is established within the first 0.8 sec. following cue onset indicating that attentional engagement was sufficient during this time period, but optimal only after after 0.8 sec following cue onset.

Supplementary Information SI 2.

Clustering-strength based on nearest neighbor distances. We quantified the distribution of nearest-neighbor distances to test whether neurons showing significant attentional modulation were more likely recorded at similar locations on the flat map compared to the null hypothesis of a random spatial distribution of significant effects. For every significant and non-significant neuron, we computed the spatial, Euclidean distance to the closest neuron with a non-matching significance, i.e. the closest neuron that was non-significant (for a significant reference neuron) and significant (for a non-significant reference neuron), respectively. This rendered a distribution of spatial distances between all neurons and their respective spatially closest neuron with a non-matching significance. The mean of this distribution was defined as the cluster coefficient. If significant attention effects are clustered in space, then this cluster coefficient should be larger than by probability. To test this, we compared the average spatial distance

against a randomization distribution of cluster coefficients, which was obtained by computing the spatial distances 1000 times with randomly interchanged locations of the recorded neurons. Cluster coefficients were z-transformed allowing to identify significant clustering of attentional effects for those time epochs where the a z-normalized coefficient exceeded $z = 1.64$ of the normalized random distribution, corresponding to a one-sided test with $p \leq 0.05$.

We tested, for each time point from -0.25 up to 1.5 sec. relative to attention cue onset, whether the significant (*ANOVA F-Test, $p<0.05$)* spatial attention and value-selectivity effects of neurons clustered in space. In addition to the mutual information in the manuscript, we used nearest-neighbor distances in order to test whether neurons showing a significant spatial attention or value-selectivity effect were more likely to be recorded at similar locations on the flat map compared to the null hypothesis of a random, uniform spatial distribution of spatial attention effects. Supplementary Figure S2 shows the attention cue aligned evolution of the normalized cluster coefficients (*see* Material and Methods for details), illustrating a rise of spatial clustering following cue onset and a significant spatial clustering at around 0.4 sec. for both spatial attention and value condition after cue onset ($p \leq 0.05$, T-test). The found patterns show strong overlap with the temporal evolution of the mutual information pattern, however the mutual information analysis offers a more sensitive approach because it takes into account the complete spatial distribution of selectivities, instead of a limited number of pairwise relationships between nearest neighbors.
